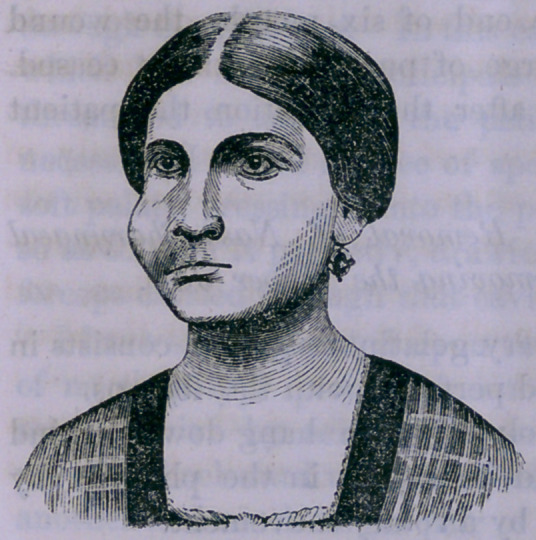# Cases of Re-section of the Upper Jaw

**Published:** 1860-04

**Authors:** Daniel S. Brainard


### ARTICLE 6.

### CASES OF RE-SECTION OF THE UPPER JAW,

### WITH SUGGESTIONS OF AN IMPROVEMENT IN THE OPERATION OF REMOVAL OF NASO-PHARYNGEAL POLYPI.

BY DANIEL BRAINARD, M. D.

Re-section of the upper jaw is not so rare an operation as to attract very great attention at the present day. It is, however, an operation of recent date, although parts of the jaw and in cases of necrosis, even the whole, were long since taken away. The practicability of re-secting it for disease is said to have been first demonstrated by Gensoul, of Lyons, and its removal first effected by Dupuytren. Even the whole of it, on both sides, has been successfully removed by Maisonneuue, Heyfelder, Tarefi, Lizzars, Liston, and others. In this country, the upper jaw has been removed by Warren, Mott,

Stevens, Eve, Mutter, Rogers, Mussey, Pancoast, Jamison, and probably by many others.

I have removed the whole of the upper jaw, on one side, including the malar and part of the palate bones, three times, and parts of it several times. The first operation was performed May 23, 1846, and published in the medical journals of the time. The tumor was fibrous and cartilaginous, probably not malignant, as the patient died about two years after, without return of the disease. The second will be reported herein.

The circumstances requiring the operation are well known, excepting as regards the case of polypus of the nostril, of large size, and extending into the pharynx, for which the removal of the jaw has been performed, but concerning which the practice cannot be considered settled. Flaubert, Michaux, Robert, Maisonneuue, Davigne, and Arrachart, have resorted to the practice. Dr. Mott laid open the nostril in a similar case, and by cutting away the anterior bony margin of that cavity was able to arrive upon the root of the polypus. Roux also resorted to incision of the nostril, but Gerdy, in the discussion at the Society of Surgery in 1852, questioned the propriety of these operations, and asserted that by repeated attempts naso-pharyngeal polypi can be removed without them. Mr. Nelaton incised the veil of the soft palate in order to arrive upon the root of a polypus.

Prof. Laugenbeck, of Berlin, has lately performed an operation "which consists in re-secting the nasal bones and the orbitar process of the superior maxillary bone, leaving a bridge of periosterum by which they may remain adherent to the neighboring parts and which permits them to re-unite after the polypus has been removed and the bones replaced. Prof. Laugenbeck has performed this operation with perfect success." (*Med. News and Library*, for March, 1860.

M. Legouest incised both the hard and soft palate. *Gazette Des Hopiteaux*, 1859, p. 168.

I add to this paper a note of some cases of this kind in which polypus was removed without incision.

Case 1.—Removal of the upper jaw for cancerous disease:

Caleb Inman, of Wisconsin, a farmer, aged 60 years, consulted me October 17, 1851, on account of a tumor of the left upper jaw. It projected in front so as to elevate the cheek—downward into the mouth where there were openings caused by the falling out of the teeth, from which pus and serum discharged—encroached upon the nostril, from which there was a discharge of mucous and projected externally beneath the zygomatic arch.

The disease had commenced about six months previously by a discharge from the nostril. Soon after a tumor appeared beneath the eye, which ruptured or was opened. Its growth had been rapid and attended with much pain. The general health was much impaired. He had been treated with iodide potass. internally, and applications of nit. silver locally.

The operation for its removal was performed October 18, 1851, in presence of Drs. Rutter, McArthur, and several other physicians and students.

The steps of the operation need not be described, as they were the same in every respect as those described in the case Miss Derey, already referred to.

The points of importance, however, to which I would call attention are these:

1. Uncovering the tumor freely by an incision extending from the middle of the upper lip to near the internal angle of the eye, and another at right angles with this, extending over the zygoma. Where the whole jaw is to be removed these free incisions are required. Where pieces only are to be taken away, it may be done through the mouth alone.

2. Dividing the palate portion of the superior maxillary and palate bones with a metacarpal saw and the nasal process, the connection of the malar to the external angular process, and the zygoma with a pair of strong bone scissors.

3. Driving a chisel, with a mallet, into the temporal fossa, and using it as a lever to start the jaw from its connections. This is only required when the tumor has a certain degree of firmness.

4. Putting upon the cheeks, from the angle of the mouth backward, the forceps with slide for controlling hemor-

Fig. 1.

rhage, already described in this journal, a figure of which is re-produced here, representing the instrument of half size. (Fig. 1.)

I have used these forceps recently, in an operation for the removal of the upper jaw, and in two very severe operations for deformity of the face, and found in all, that it controlled the circulation in the facial artery perfectly.

The attachments of the soft parts can then be readily divided with the bistoury.

This plan permits the greatest dispatch, which, when there is much hemorrhage, as was the case in this instance, is an essential point.

The patient was kept upon stimulants and a nourishing diet and at the end of three weeks was able to return home with the wound nearly healed.

The tumor was soft and the antrum found filled with a fungus growth. For four years there was no return of the disease, but at the end of that time the growth re-appeared, and soon caused his death.

Case 2.—Mrs. Rogers, of Bureau Co., Ill., aged 20 years, was sent to me by Dr. Hopkins, of Princeton, in March, on account of a tumor of the right upper jaw. She stated that about two years since, she had a tooth that was troublesome, and which she dug out herself about a year ago, March, 1859. Soon after, a fungus began to grow from the socket which was repressed in some degree by caustic. This proving insufficient, it was removed by operation, August, 1859, soon after, the fungus re-appeared and was again removed by operation, Nov., 1859, this time the wound cicatrized, and for about three months no return was noticed. In Feb., 1860 it grew again so rapidly, that March 5th it was of the size of a small orange, projecting in form of a bleeding fungus into the mouth. The external appearance is shown in the figure. This patient is young and vigorous, and her health has suffered but little, although recently she has taken morphia to obtain rest at night.

It was removed March 7, 1860, with the assistance of Drs. Wheeler, Powell, Durham and several students, in the manner already described. The spongy part of the ethmoid bone and the vomer were so connected with the diseased mass as to render their removal advisable.

The tumor was distinctly encephaloid in structure, and under the microscope, presented the appearance described as that of cancer cells, in a marked degree.

No dangerous symptoms followed, and at the end of two weeks the patient was able to walk about and take her usual food.

Case 3. Bony cyst of the anterior wall of the antrum removed:

Mrs. R., aged 34 years, of good constitution, consulted me, in the summer of 1858, on account of a projection of the left cheek, presenting a projection of the size of a large nut. It was of bony hardness, tender to the touch.

She stated that this swelling made its appearance twelve years previously, when it was soft and free from pain. It gradually acquired a bony hardness. In December, 1857, it suddenly became painful, the pain extending over the whole of that side of the face and head. After this had continued about two weeks she felt a sensation of something "giving way," which was followed by a copious discharge of pus from the nostril, which had continued up to this time.

This tumor was removed by an incision through the cheek. A bony cyst of the size of a hickory nut being cut away with the chisel and hammer. In the side next the antrum was an opening through which the pus had discharged itself. A tent was kept in the wound for three weeks, when it was withdrawn. At the end of six months the wound was healed and the discharge of pus had entirely ceased. Recently, more than a year after the operation the patient remained quite well.

*Suggestions Concerning the Removal of Naso-Pharyngeal Polypi, without Removing the Upper Jaw.*

1. The treatment of ordinary gelatinous polypi, consists in extracting them carefully and perfectly with the forceps.

2. Fleshy and Fibrous polypi, which hang down behind the veil of the palate, should be seized in the pharynx by strong forceps, and removed by a rotary movement.

3. Vascular polypi may be crushed and left *in situ*, when they disappear.

These propositions will, I think, hardly be questioned, for putting a ligature around a polypus which sinks behind the veil of the palate, is unnecessary; if it remains above it, it is very difficult, and often impossible.

But there remains the question of the treatment of those large, firm polypi, situated deeply at the back part of the nostril, which cannot be drawn forward, and which do not project sufficiently behind the palate to admit of being seized with ordinary forceps, and for which the operation of removal of the upper jaw, an incision of the nostril or of the soft or hard palate, has been proposed and performed by Mr. Manne, in 1847, and by M. Maisonneuue, after what he terms the method of the *boutonniere*, viz: by an opening in, instead of division of the edge of the veil of the palate.

In 1841 I attempted to remove a large fleshy polypus with the forceps, which resisted all my efforts, although it was seized repeatedly, and torn. By passing the finger behind the veil of the palate for the purpose of exploration, which can be readily done, I found a body so large projecting beyond the posterior nasal orifice, into the upper part of the pharynx that it was at once evident that it could never pass through the nostril. I then seized it as firmly as possible with the polypus forceps, and instead of pulling it forward, thrust it backward, when its attachments were broken, and it was removed through the pharynx, In this case a gush of blood followed, which had not been anticipated, and which, for a moment, threatened to suffocate the patient. Under the stimulus of necessity, I seized a piece of sponge and passed it behind the soft palate, pressing it into the posterior orifice of the nostril, so as to plug it perfectly, drawing it into the nostril with the forceps carried through that cavity.

I have since operated upon two cases of the severest forms of nasal polypi projecting into the pharynx, in a similar manner, viz: by seizing the anterior part with forceps, and pressing backward until the back part could be reached with another and much larger pair of curved forceps, with which it was firmly grasped and torn away by a gradual but firm rotary movement. In one of these cases the polypus immediately after removal weighed eight ounces. The hemorrhage was terrific but was stopped by filling the nostril from behind with pieces of sponge. This method of plugging the nostril seems to be little known, but is not difficult to accomplish in the adult, in the natural state of the parts. When the pharynx has become accustomed to the presence of a polypus, and the soft palate has been pushed downward and forward, nothing can be easier.

This is a point of importance, for the motive which induced the older surgeons to adhere to the ligature, was undoubtedly the fear of hemorrhage. The only danger which need be apprehended from the use of sponge, in this way, is that of its falling into the air passages, as happened in a case reported by Prof. Peaslee, in which, however, the sponge was, I think, introduced from before.

By this means I am sure that some polypi may be safely extracted which otherwise resist all attempts, unless the jaw itself, or the soft or hard palate, or both, be divided. I do not, however, assert that this is always the case, since the extent of the attachment, and its firmness, might offer too great resistance. If this should be found, on trial, there still remains the method resorted to by professor Letenneur, of the School of Medicine of Nantes, who, in a case of this kind, having failed to pass a ligature around the pedicle, resorted to the use of forceps similar to the *enterotome* of Dupuytren, and by fixing it successively upon different portions with the screw, each in turn was destroyed, the pedicle being at last seized. The dangers incident to the removal of the upper jaw, and even to the incision of the palate by the method of the *boutonniere* are sufficient to induce the surgeon, before proceeding to the application of either of these methods, to use with care all the resources of art for the purpose of effecting his object in a manner less severe. It is probable that more perfect instruments may yet be produced, which will render this object of more easy attainment.

## Figures and Tables

**Fig. 1. f1:**
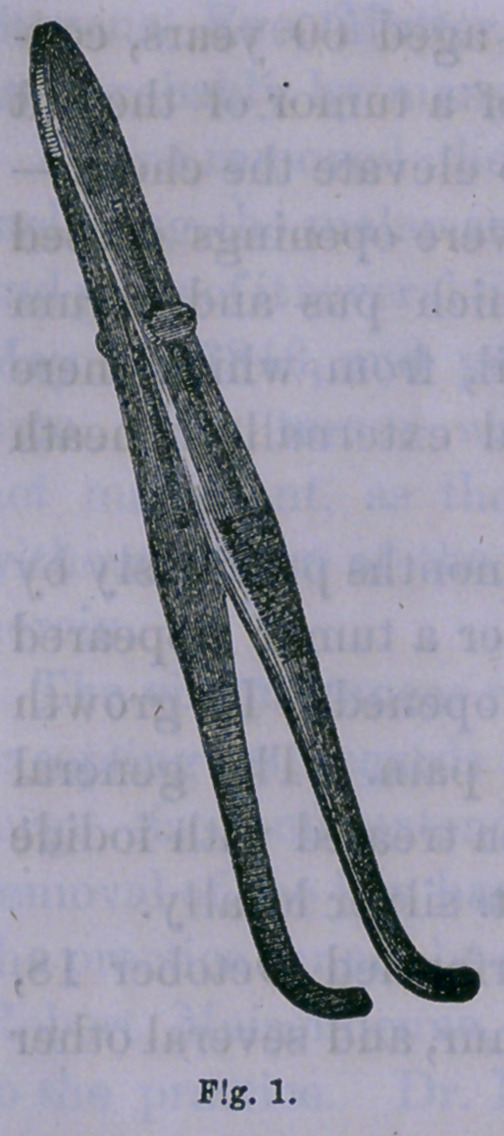


**Figure f2:**